# An innovative team-based stop smoking competition among Māori and Pacific Island smokers: rationale and method for the study and its evaluation

**DOI:** 10.1186/1471-2458-13-1228

**Published:** 2013-12-23

**Authors:** Marewa Glover, Amber Bosman, Annemarie Wagemakers, Anette Kira, Chris Paton, Nathan Cowie

**Affiliations:** 1 Centre for Tobacco Control Research, Social and Community Health, University of Auckland, Auckland, New Zealand; 2 Master Health and Society, Wageningen University, Wageningen, Netherlands; 3 Health and Society Group, Department of Social Sciences, Wageningen University, Wageningen, Netherlands; 4 George Institute, University of Oxford, Oxford, UK

**Keywords:** MeSH terms: health promotion, Smoking cessation, Indigenous, New Zealand, Others: competition, Ethnic minorities, Indigenous smoking

## Abstract

**Background:**

Māori and Pacific Island people have significantly higher smoking rates compared to the rest of the New Zealand population. The main aim of this paper is to describe how knowledge of Indigenous people’s practices and principles can be combined with proven effective smoking cessation support into a cessation intervention appropriate for Indigenous people.

**Methods/Design:**

A literature review was conducted to identify what cultural principles and practices could be used to increase salience, and what competition elements could have an impact on efficacy of smoking cessation. The identified elements were incorporated into the design of a cessation intervention.

**Discussion:**

Cultural practices incorporated into the intervention include having a holistic family or group-centred focus, inter-group competitiveness, fundraising and ritual pledging. Competition elements included are social support, pharmacotherapy use, cash prize incentives and the use of a dedicated website and iPad application. A pre-test post-test will be combined with process evaluation to evaluate if the competition results in triggering mass-quitting, utilisation of pharmacotherapy and in increasing sustained smoking cessation and to get a comprehensive understanding of the way in which they contribute to the effect. The present study is the first to describe how knowledge about cultural practices and principles can be combined with proven cessation support into a smoking cessation contest. The findings from this study are promising and further more rigorous testing is warranted.

## Background

Tobacco kills nearly 6 million people worldwide, and causes hundreds of billions of dollars of economic damage each year [1]. New Zealand (NZ) has had a progressive tobacco control programme since 1994 with sustained strong tobacco control policies, for example smoke-free environments, increasing tax on tobacco, and public health programmes, and since 2000 an increasing range of cessation support, such as, the national Quitline [2]. This comprehensive programme has led to a drop in daily smoking prevalence from 26.6% in 1997 to 19.2% in 2009 [3,4]. However, each year 4500-5000 people are still killed by smoking or exposure to second-hand smoke [3]. Considerable inequity exists across the population with smoking prevalence among Māori at 45.1% and Pacific Island people at 30.3% compared to New Zealand European at 19.7% rates [3].

The government of New Zealand has set an ambitious goal for the country to be smokefree, defined as “a smoking prevalence of less than 5%, with tobacco being difficult to obtain and children not exposed to smoking”, by 2025. To achieve this, new and innovative strategies are needed to bring about rapid reduction in smoking prevalence. Generic population tobacco control programmes, however, have increased the gap between different SES groups and ethnic groups [5]. Therefore there is a need for programmes specifically focused on disadvantaged population groups.

Interventions that have shown promise internationally are stop smoking competitions where usually individuals who smoke stop for a chance to win a prize of cash or products. Quit and win competitions have run all over the world since the early 1980s [6] with long term quit rates ranging from 8% to 20% at 12-months [7]. Short term quit rates at the end of the competition have been as high as 66% [8]. Only a few quit and win competitions have focused on disadvantaged population groups. However, one quit and win competition aimed at low-income smokers in the United States had significantly higher overall quit rates in the treatment group with 23% (compared to 9% in the control group) still quit at 12-months [9]. Māori and Pacific Island people in NZ are overrepresented among lower socio-economic groups [10,11]. A quit and win competition was piloted in 2000 in the Hawke’s Bay region of New Zealand and achieved quit rates of 40% at 12 months follow-up [12]. The competition included a high proportion of Māori (32% compared to 22% of the total Hawkes Bay population).

Given NZ’s heightened urgency to increase quitting on a mass scale, and the positive potential for quit and win competitions to be attractive to and more effective at bringing about cessation among smokers from lower socio-economic groups it is time to test quit and win competitions for Māori and Pacific Island people [13]. We propose a quit and win competition which is aligned more with Indigenous people’ cultural principles and beliefs. The main aim of this paper is to describe how knowledge of Indigenous people’s practices and principles can be incorporated with proven effective smoking cessation support into a culturally appropriate cessation intervention WERO (WERO in the Māori language means challenge). Included in the aim is describing identified cultural principles and practices that can be used in a cessation competition and describing identified effective cessation elements that could be incorporated. A final aim of the paper is to describe the WERO study protocol.

### Cultural principles and practices

Designing public health interventions consistent with the cultural beliefs and practices of target populations is believed to lead to better receptivity, acceptance and salience of health information and programs [14].

#### Competitiveness

The indigenous people of NZ are collectively called Māori but there are many tribes within the Māori nation. Tribal identity is strong and historically marked by inter-tribal rivalry and competition, which carries on in modern times [15,16]. This rivalry and competition is not a means of excluding others, rather it develops and maintains a sense of collective unity, loyalty and belonging within the group [16]. Kapa haka (traditional Māori performing arts) regional and national competitions are an important way to construct a secure Māori identity and the skills learned can be transferred into other areas of life [17]. Māori and Pacific Island peoples have higher participation rates in sport, recreation and physical activities than other ethnic groups in NZ [18]. For instance, kapa haka contests are well-attended and popular. Te Matatini National Festival which is held every two years attracts up to 30,000 participants and visitors [19]. Other group competitions include Top Town, where communities compete for their town, and inter-marae (traditional tribal meeting place) sports events, to celebrate family values, strengthen kinship and assist in maintaining cultural values and knowledge in an atmosphere of friendly competition and healthy lifestyles [20]. Among Pacific Island people, competitive sports such as kilikiti (a Samoan form of cricket) are popular, with high participation rates [21]. These contests incorporate competitiveness with cultural elements and encourage community participation, all of which are key components for effective health interventions for Indigenous people [22]. Furthermore, competitions have also been used to encourage behaviour changes, such as weight loss (for example [23]) and seatbelt usage among teenagers (for example [24]).

#### Family centred

Like many other Indigenous people, Māori and Pacific Island peoples are whānau (family) centred, where the wellbeing of the family, church, extended family and culture is often placed above personal health. The NZ government *Whānau Ora* policy was developed to establish and recognise the centrality of the family in the delivery of health and social services. Recently, health provider organisations with a sole focus on Pacific Island peoples have been included in the *Whānau Ora* programme [25]. *Whānau Ora* is an inclusive approach to providing health services to families across New Zealand. It builds on the strengths and capabilities present in families as a whole, rather than focusing separately on individual members and their problems [25,26].

A smoker’s motivation to smoke is affected by environmental, social, economic and personal determinants [27]. Māori smoking cessation services provide support to individuals and families to quit together. Support is typically extended to other smokers in the presenting individuals’ friendship network, household or work environment [28]. Group cessation approaches therefore align with Māori and Pacific Island whānau centred values and practices and increase the number of smokers assisted at a time.

#### Fundraising

Another common Indigenous culturally embedded practice is fundraising. Both Māori and Pacific Island cultures organise and participate in fundraising for schools, churches, cultural events and competitions, sports, marae and family reunions [29]. Voluntary activity, fundraising, is for many Māori and Pacific Island people an act of service to their whānau, and iwi (tribe) and one way of fulfilling family and social obligations and responsibilities. This activity is central to identity and to the maintenance of culture and traditions. In 2001, Māori were more likely than non-Māori to have been involved in unpaid activities outside the household [30]. Many Pacific Island people see volunteering as a moral and ethical responsibility, and a cultural duty that is passed on through traditional roles and responsibilities to the collective group and their family [30,31].

### Effective elements of smoking cessation competitions

Most quit and win contests include a cessation support programme in addition to the contest [32]. A few quit and win studies have investigated the effectiveness of general cessation support, such as pharmacological support, social support, use of incentives, and technology, in a quit and win context [33-36]. Elements of a smoking cessation competition are considered effective when they significantly impact the chances of short-term and/or long-term abstinence.

#### Pharmacological support

In general, the use of any type of pharmacological support is likely to increase cessation rates [32,33,37-39]. Nicotine Replacement Therapy (NRT) increases the chances of successfully stopping smoking by 50% to 70% [39]. Similar findings have been found for Māori: smokers who took bupropion doubled their cessation rates in comparison with the placebo group [40].

When comparing NRT use with or without competition, Maheu et al. [41] found that combining contest and NRT led to a significant increase in number of smokefree weeks, in comparison with NRT without contest. However, another study found that there was no significant long-term difference in cessation rates between those quitters who used no pharmacological support and those who did [8].

#### Social support

Social support, given by family, friends and co-workers, has a positive influence on smoking cessation in general [42,43] and has a particularly significant impact when combined with quit and win competitions [33,34,37,44,45]. Cessation rates are higher for contestants with a designated support person compared with those without [34,44]. One study found that 60% of their contestants utilised a designated support person, which reflects the importance of this type of support [44]. The type of relationship that the support person had with their smoker has an influence as well where having a spouse or partner as a buddy was most effective [37].

#### Incentives

Cash incentives have been shown to boost motivation to quit [45,46]. Although the effects on long-term abstinence are not yet clear, there is a short-term effect [32]. For example, in one study 57% of the participants stated that the chance of winning a cash prize had helped them to remain abstinent [46].

#### Technology to support quitting

There has been a rise in use of e-Health, such as internet, smartphone and tablet PC applications (apps), to prompt and support behaviour change to improve health. Such technologies have the potential to reach large numbers of people [47,48]. Internet-based social support groups can connect people in disparate locations and can provide support at all times of the day, at a minimal cost [47,49]. Often posts on website message boards allow quick responses by peers, indicating that social networking may be beneficial to prevent relapse by smokers trying to quit, especially in comparison to the time it may take to seek professional help [49].

In the area of smoking cessation, the internet has provided a platform for interactive support. One such social network for tobacco users, QuitNet, has a large number of members who persist over time, even after quitting. This shows that social networking may prevent relapse for a lengthy period of time [48]. The use of the internet for smoking cessation is most effective when there is a high frequency of visits to the website. A randomised controlled trial which tested the effectiveness of internet use, in conjunction with other smoking cessation interventions such as NRT, found that the number of times participants used the website per week was significantly related to abstinence both at the end of the treatment period and at the 6 month follow up; yet purely access to the website was not [50]. Smartphone (mobile phones which can also run computer programmes or apps) and tablet PC apps have also been used to assist with smoking cessation. However, while there is a large amount of research about the use of other types of technology to promote behaviour change, there is very little research in this area, given how recently apps have been introduced.

### Integrating cultural and smoking cessation elements into the WERO intervention

Incorporating Māori and Pacific Island people’s inherent interest in competition into a cessation intervention could potentially increase attractiveness of the intervention and thereby increase uptake and retention. However, to the best of our knowledge, no Indigenous-informed quit and win contest has been trialled and published. The vast majority of Quit and Win competitions pitch individuals against each other [7]. But because of the strong family-community centrality of many Indigenous peoples, group-, rather than individual-, based competition may be more appropriate. Therefore, the main part of the intervention was an inter-team contest where the team with the most quitters at end of competition won a prize.

In order to increase the effectiveness of the contest, other cultural and smoking cessation elements were also included. A contest that ties in with Māori and Pacific Island people’s practises of fundraising may be attractive. Benefitting their community may be a stronger motivator to quit than individual benefit. Therefore, in WERO, the winning prize did not go to the team participants, but rather an organisation or charity of their choice. This fundraising aspect is expected to appeal to participants’ existing sense of duty to raise funds for cultural or local community events and other needs. This may provide additional motivation over and above any motivation to quit for their own benefit.

Further, members of each team had their progress displayed on a public website. Lastly, given the substantial and well documented increase in quit success with the use of pharmacological support, these were also included in the intervention.

#### WERO intervention

WERO participants compete in teams of ten current smokers, the majority of which knew each other prior to the intervention, which boosts peer social support and enable the dynamics of whanaungatanga (a relationship through familial and shared experiences) to operate. Each team appoints a ‘coach’ whose role is to encourage their team and facilitate team meetings and activities. Criteria for the coaches are that they are non-smokers or ex-smokers, and that they are willing to support their team to quit over the contest period.

The team with the most smokefree members at the end of the competition wins. If multiple teams have the same number of smokefree team members, one winner will be randomly drawn by a computer random digit generator. The prize of $5000 goes to their nominated charity or community organisation. If a team member tries to cheat the competition, team members would continue to receive support to quit but they would be excluded from the prize draw.

WERO participants are encouraged and supported to use NRT, such as, nicotine patches, gum or lozenges, which in New Zealand can be obtained using a Quitcard for as little $5 for 8 weeks supply. They are also told about Champix (varenicline) and Zyban (bupropion) which are subsidised, but only available on prescription. The *New Zealand smoking cessation guidelines* recommend NRT be used for a course of at least 8 weeks [51], thus the WERO competition period will run for 3 months.

An interactive website and iPad app is used to strengthen the sense of team and inter-team competitiveness by making the smoking status of team members and the team as a whole publicly visible. To keep all teams up-to-date on their own and other team’s performances, weekly updates of smoking status are requested from the regional Coordinators. Teams and supporters are also able to post questions, comments or support, to the team page. Frequent interaction with the website should help reduce relapse, and will be encouraged with the use of a prize of $500 for the team that utilises their team page the most. In addition, the website and app provides information in the style of tips on quitting and relapse prevention. Participants’ questions or comments about withdrawal symptoms and trigger situations on their web page can also be answered and commented on by the study’s primary investigator, an expert in smoking cessation.

Although it would be preferable to develop the app for multiple operating systems (OS), in the interests of cost-containment it was decided to develop for one OS and form-factor for this pilot. Further, the study team did not have the capacity to test the performance of an app across the wide range of tablets available. The iPad was chosen over other types of tablets as it has a wide user base and high levels of usability compared to other tablet PC operating systems such as Windows and Android.

## Methods/Design

WERO is one project within a broader programme of research being conducted or commissioned by the New Zealand Tobacco Control Research Tūranga. The Tūranga (platform) aims to identify innovative interventions and policies that could dramatically increase quit rates to speed NZ towards Smokefree 2025, whilst also decreasing inequities in smoking prevalence between Māori and Pacific Island people and non-Māori non-Pacific Island populations. Tūranga projects are expected to produce results quickly thus limiting study design. Randomized controlled trials and in-depth cost-benefit analysis for instance are beyond the budget and time constraints of the Tūranga. Thus, WERO will be evaluated using a multi-method approach consisting of a pre-test post-test design to assess effectiveness and a process evaluation to assess the contribution of the included effective elements in the programme and participants’ progress and motivation.

### Locations

WERO will be trialled in three regions: in rural Northland, in an urban Auckland Māori community and in the urban Auckland Pacific Island community. Running WERO in three different communities allows for testing for fit and effectiveness in different contexts (urban, rural) and for different ethnicities (Māori and Pacific Island people). The communities were chosen for their large proportion of Māori or Pacific Island people with high smoking prevalence, and for the availability of support services, like community health workers and smoking cessation providers.

### Regional coordinators

WERO co-ordinators, an identified person at regional level who can facilitate local use of WERO, will be appointed for each region. A District Health Board (DHB) healthy lifestyles team have been subcontracted to promote WERO and recruit teams in Northland. An urban Auckland Māori authority Whānau Ora provider have been subcontracted to deliver WERO to their urban population, and a Pacific Island WERO Co-ordinator was employed to work with Pacific Island churches. In addition to implementing the intervention, the WERO co-ordinators will also act as a research assistant and collect data.

### Team participants

Fifteen teams of ten Māori or Pacific Island smokers, total of 150, participated in this study. There were five teams from each region.

### Team coaches

In addition there were 15 coaches. The team coaches were not part of the team, but where selected, in addition to the team, in a support role.

### Inclusion criteria for research

Self-identified smokers aged 18 years and over and willing to take part in the research were eligible to participate. Smoking status was validated using exhaled carbon monoxide (CO) and to be eligible to enter, participants needed to have a baseline CO rating greater than 6 ppm.

### Exclusion criteria for research

People, who would not provide written consent for their participation, non-smokers or very light/infrequent smokers whose exhaled carbon monoxide (CO) was less than 7 ppm at entry, were excluded from the study. To ensure the competition was being tested with smokers, biochemical verification at entry was thought desirable, thus very light or infrequent smokers would likely be excluded.

### Power calculations

Assuming 80% power, 5% significance, 1-sided test, no design effect, and a standard treatment 3 month quit rate of 24% (from the National Quitline), the study would need approximately 100 persons total to detect a target 3 month quit-rate of 35%. However, because the drop-out rate at follow-up is assumed to be high (50% from past studies), 150 smokers were recruited.

### Measures

The main outcome will be biochemically validated smoking status at the end of the competition. Main analyses will be carried out on an intention-to-treat basis. Participants lost to follow-up will be assumed to be smoking. Since quit and win can have high rates of deception, smoking status will be assessed using CO tests pre and post contest. CO tests were chosen for pragmatic reasons, namely its low cost, ease of use and its availability for healthcare providers. It is limited however to detection of active smoking within the previous 2-5 hours only [52], but unfortunately other methods are cost-prohibitive [53]. The CO tests were used to determine which team will win the prize. Secondary outcomes will be self-reported point prevalence at 6 months follow-up, use of pharmacotherapy, duration until relapse, tobacco consumption, and activity on the website. Because there will not be any incentives for participants’ to be smokefree at 6-months follow-up, the risk of deception is lower than at end of competition. Therefore, self-reports was considered sufficient. Furthermore, not doing a CO test at 6 months was also about reducing research burden for participants and research assistants, and using self-report enabled follow-up to be conducted by telephone.

At baseline and 3 months follow up exhaled CO will be measured using a Bedfont Smokerlyzer CO monitor. The amount of CO in ‘end-expired air’ is a good approximation for the concentration of CO in the blood and exhaled CO levels will be higher in smokers than in non-smokers. There is however no agreement about what the optimal cut-off point is. Middleton and Morice [54] found a cutoff level of 6 ppm, which detected 94% of smokers and 96% of non-smokers in a respiratory outpatient clinic. In this study a relatively low cutoff point of 6 ppm will be used, because there is low air pollution in New Zealand [55].

At baseline, 3 and 6 months participants will be asked to complete paper-based questionnaires with 33 close-ended questions (Table 1). These questionnaires are used to determine the progress of the participants and to determine the short-term effects of the competition.

**Table 1:** Questionnaire topics

**Category**	**Topic realms**
Demographic	Sex, ethnicity, age, marital status, education level, community service card (proxy for socioeconomic status)
Smoking behavior	Smoking status, amount smoked, time to first cigarette, previous cessation attempts and methods used.
Quitting behavior	Number of quit attempts, any support used to quit, reason for quitting and interest in cessation support
Others smoking	Presence of smokers in participants’ social networks? Does anyone else in the house smoke? Do people smoke indoors?
WERO – related	Reasons for stopping during WERO? Cessation methods of interest? Relationship with other team members? Competition methods that help team smoking cessation? Social support by family and friends.
Technology	Access to mobile phone, IPad/tablet, Internet? Use of social networking sites.

### Process evaluation

To get insight into the efficacy of WERO, qualitative interviews will be conducted with coaches and the WERO coordinators at the completion of the 3 month data collection. The interviews will include questions on intervention implementation, acceptability and satisfaction with the intervention, identification of barriers for effective implementation, and suggestions for improvement.

### Ethics

Ethics approval for this study was granted by the HDEC Northern X Regional Ethics Committee for 3 years on 5 December 2011 (ref. NTX/11/EXP/308).

## Discussion

Quit and win competitions have the potential to be attractive and effective in New Zealand. Previous competitions that targeted disadvantaged smokers did not incorporate cultural elements to increase the attractiveness or relevancy for different ethnic groups in their populations [9,19]. Aligning cultural characteristics of target populations with public health interventions is believed to lead to better receptivity, acceptance and salience of health information and programs [14]. The present study is the first to include multiple cultural elements in a smoking cessation contest. This project adopts the competition approach to prompt quit attempts among Māori and Pacific Island people. Culturally aligned adaptations include use of a team-based competition with cash prizes donated to a charity or community based organisation of the winning team’s choosing. For NZ to reach its goal of 5% or less smoking prevalence by 2025, new interventions that trigger mass quitting are needed. If effective, WERO has the potential to engage whole communities in supporting quitting. WERO will also provide information that could be useful for designing cessation interventions for other Indigenous and minority populations.

A major strength of WERO is that it is culturally aligned and salient, and therefore likely to be attractive to high priority groups – Māori and Pacific Island people. Testing the competition in rural and urban settings will also inform generalisation to different settings. Further, WERO is pragmatic in that it was designed to be delivered by existing service providers, except for the web-based technology component. If WERO is effective, it can be rolled out rapidly to other areas in New Zealand. WERO also supports building local cessation capacity among the Māori and Pacific Island population. The coaches develop skills that when the competition is finished they may use to help other people in their community stop smoking. Another strength is that a multiple methods design is used, combining qualitative and quantitative, where internal validity of qualitative data is ensured by participant check and external quality is ensured by use of multiple communities, regions and subgroups.

There are also limitations to the study. Firstly, the need to produce information for the government quickly on whether this intervention is effective prohibited the use of a RCT. Secondly, attrition poses a special problem for teams-dependant competition. However, it is hoped that the use of team support might keep attrition rates low. Thirdly, motivation to win the cash prize may motivate participants to falsify their smoking status. CO measurement at baseline and at 3 months will assist with reducing false reports. Other potential confounders, such as the presence of smokers in participants’ social networks will be measured in the questionnaire.

## Competing interests

The authors declare that they have no competing interest.

## Authors’ contribution

MG conceived of the study and was involved in all aspects of the study. AB participated in design of study, review of the literature and helped obtain ethics approval and drafted the manuscript. AW provided academic supervision for AB which involved advising on the design of the study, reviewing and critically contributing to the draft manuscript. AK participated in design of study, review of literature and critical revisions of the manuscript. NC and CP participated in design of study, website and iPad application and development of other study materials and documents. They provided input into and critical review of the manuscript. All authors read and approved the final manuscript.

## Authors’ information

MG is a leading Māori health researcher and Co-director of the New Zealand Tobacco Control Research Tūranga. AW an Assistant Professor in the Netherlands Wageningen University & Research Centre Health and Society (HSO) with expertise in community health promotion. MG and AW have an ongoing collaboration involving the placement of Dutch Interns with the Centre for Tobacco Control Research. CP is a medical doctor with significant experience in m-Health.

## Pre-publication history

The pre-publication history for this paper can be accessed here:

http://www.biomedcentral.com/1471-2458/13/1228/prepub
